# Education Recovery In Conflict-Affected Settings

**DOI:** 10.1007/s40653-026-00944-w

**Published:** 2026-07-14

**Authors:** Alison Willis

**Affiliations:** University of the Sunshine Coast, Sunshine Coast, Australia

**Keywords:** Education recovery, Learning loss, Disruption, Conflict-affected education, Learning discontinuity

## Abstract

Education recovery literature is typically positioned in developing or conflict- or crisis-affected contexts and often involves rebuilding schools and reintroducing curriculum. However, to date, the phenomenon of education recovery has not been modelled or defined in literature. Without a clear understanding of the phenomenon, resource allocation and programmatic responses lack an evidence base. Therefore, a phenomenographic methodology was employed to investigate the lived experiences and perspectives of people who had experienced education disruptions caused by political violence or war during their childhood and adolescence that affected their schooling, university and/or vocational education. Semi-structured interviews (*n* = 30) and a pilot survey (*n* = 66) were used to collect data. Findings identified outcomes of disruptions including gaps and gains in knowledge and skills (e.g. literacy and critical thinking), and personal attributes (e.g. confidence), and supports and motivations for recovery (e.g. family). The need to triage and identify root causes of disruptions was salient in the findings. The vulnerability of young adults was evident, as they report the highest negative impacts on confidence, focus, and self-efficacy. Findings show that education recovery after disruption in conflict-affected settings is a journey that is future-focused and dependent upon support.

**Supplementary Information:**

The online version contains supplementary material available at https://doi.org/10.1007/s40653-026-00944-w.

## Introduction

This paper maps and defines the phenomenon of education recovery after disruptions in conflict-affected settings. Education recovery literature often has a focus on rebuilding education systems and schools after disaster (e.g. Chanduvi et al., 2022; Frankenberg et al., 2013; Srivastava et al., 2021); however, the phenomenon of education recovery after disruption is not defined in literature. Typically, recovery efforts in conflict-affected settings are program based (e.g. Barakat et al., 2013). However, without a clear understanding of the phenomenon, resource allocation in recovery efforts is without an evidence base. What is not understood is the interrelated complexity of disruptions caused by conflict, what knowledges, skills and attributes are most adversely affected, and how people overcome education disruptions and fill learning gaps. In a response to this need, a phenomenographic methodology was developed to explore relationships between lived experiences and learning so that education recovery can be mapped, modelled and defined (see findings below). To do this, the University of the Sunshine Coast partnered with education officers and social welfare units in the Salvation Army in Southern Africa to better understand the causes and outcomes of disruptions to education, and what pedagogical supports and motivations are needed for recovery. In the context of this paper, disruption refers to circumstances that caused the cessation of education; interruptions without cessation are reported elsewhere (Willis et al., 2025).  

This phenomenographic study investigated the conceptions of lived experiences of adults who experienced education disruption during their childhood and adolescence in schooling, university and/or vocational education in South Africa and Mozambique. Southern African geography was chosen due to the multiple disruptions affecting education, including war and political violence, and subsequent sexual violence, poverty, sickness, political instability, and crime. Survey and interview mixed-methods identified how disruption affects knowledge, skills and personal attributes. The findings in this paper are part of a greater study that also investigated teaching strategies and learning experiences that were efficacious in education recovery (Willis et al., 2025). However, the focus of this paper is on the education disruptions and the supports and motivations necessary for education recovery in conflict-affected settings.

Accordingly, the following research questions were used to generate data for a phenomenographic model:

1. How do people who have experienced education disruption fill gaps and progress learning during and after disruption in conflict-affected settings?
2. How can communities be effectively resourced to recover from learning disruptions?

Of note, findings are specific to education disruption survivors rather than educational institutions as this study is an investigation of lived experiences over time. For example, schooling might have been disrupted in childhood, but recovery was made later in community groups in adulthood. Findings from an extensive qualitative and quantitative dataset are presented in this paper to map the phenomenon of education recovery, as this is currently not available in literature.

## Education Recovery in Literature

Education recovery in conflict-affected settings may be defined as a “social problem” according to Spector and Kitsuse’s (2017) definition: “unwanted, unjust, immoral or harmful [social conditions] that should be addressed” (p. 185). The concept of a social problem shifts the focus of recovery to systemic responsibility, rather than individual deficit. The phenomenon of education recovery is often most visible after a crisis (e.g. war, pandemic or natural disaster). Seeger et al. (1998) define a crisis as: “a specific and non-routine event or series of events that create high levels of uncertainty and threaten or are perceived to threaten life and property or general well-being” (p. 233). A definition for education recovery could not be found in literature; therefore, this study endeavours to make a contribution in this regard for the sake of the millions of children and adolescents affected by war around the world. Conflict increases the mental health burden in a society, negatively impacting cognitive function, attention and emotional regulation (Aqtam 2025; Aqtam et al. 2025a).

Quality Education is the UNESCO Sustainable Development Goal 4 (SDG4), which includes “ensuring inclusive and equitable quality education and promote lifelong learning opportunities for all” (United Nations, n.d). It is acknowledged by the United Nations that SDG4 is an enabler of most other SDGs. Due to COVID-19, socioeconomic disparities and other hijacking factors, meeting SDG4 targets has been a considerable challenge. Further, education recovery is a priority of UNICEF, evident in the development of a five-step approach (RAPID) for schools to recover education after COVID-19 school closures (Chanduvi et al., 2022). RAPID comprises: “**r**each every child and retain them in school; **a**ssess learning levels; **p**rioritise teaching the fundamentals; **i**ncrease catch-up learning and progress beyond what was lost; and **d**evelop psychosocial health and well-being so every child is ready to learn” (2022, p. 5). Of note, RAPID is a recovery strategy, and in conflict-affected settings attending to welfare needs (security, housing, food) may need to be tended to first.

COVID-related learning opportunity losses highlighted inequities in education systems around the world, as school closures had greater effects on students in three particular demographic categories: lower-socioeconomic backgrounds, lower educated parents, and from marginalised groups (Srivastava et al., 2021). Global modelling using data from 205 countries has indicated that COVID-19-related learning losses could convert to losses in lifetime earnings of US$15.3 trillion (Psacharopoulos et al., 2021). Engzell et al. (2021) measured significant declines in results in standardised national testing due to the pandemic but also admit that the full extent of learning losses are unknown. Notwithstanding, it must also be acknowledged that learning gains (in literacy, numeracy and other areas) were also made by some students during the pandemic (Chen & Krieger, 2023; Donnelly & Patrinos, 2022).

Additionally, education recovery literature also investigates economic losses due to other education disruptions (e.g. political coups in Chand, 2000; natural disaster in Opabola & Galasso, 2024). Education recovery literature has a strong focus on rebuilding systems and infrastructure after disaster (e.g. Chanduvi et al., 2022; Frankenberg et al., 2013; Srivastava et al., 2021). Education recovery often occurs parallel to housing recovery as was seen in the Philippines after the 2013 typhoon (Maly & Sakurai, 2023), and literature shows that housing security was a protective factor after the 2004 tsunami in Sumatra, Indonesia against education disruptions and associated poverty and child labour (Frankenberg et al., 2013). Both in the literature and in the greater findings from this study, there is an evident reciprocal relationship between infrastructure security and education – that is, infrastructure insecurity can lead to education disruption, and education disruption can perpetuate poverty and infrastructure insecurities. Therefore, this study took an interest in infrastructure insecurities when investigating education disruptions.

A previous education disruption and recovery study conducted in conflict-affected Northern Uganda demonstrated how teachers played an important role in helping students overcome the effects of stress and trauma on learning. Where students had suffered the effects of displacement, tragic loss and trauma, and it was found that teachers played a pivotal role in their neurological, psychological and sociological rehabilitation (Willis & Nagel, 2015). Recent studies in Ethiopia and Palestine highlight the profound damage of war on education attainment and the realisation of SDG 4 due to social, psychological and cultural wounds (Gebre-eyesus & Reda, 2024; Aqtam et al. 2025c).

In contexts where education is used to perpetuate radicalism and violence, education has been effective in providing solutions for peace (e.g. in Sibolga Indonesia in Dasapong et al., 2023). Notwithstanding, school-related gender-based violence is exacerbated in conflict and crisis situations. For example, a case study in Mozambique reported 47% of students stating sexual violence occurs in schools (United Nations Girls Education Initiative (UNGEI), 2024).

The hijacking effects of stress and trauma on learning are well established in literature (e.g. Aqtam et al. 2025b; Cozolino, 2014; Gebre-eyesus & Reda, 2024; Willis et al., 2021). So too are the effects of poverty: for example, pandemic-related learning losses were most pronounced amongst students of low-socioeconomic status (e.g. Engzell et al., 2021 in the Netherlands; Landa et al., 2021 in South Africa), and it is more difficult for poor schools to overcome inherited socio-economic disadvantage (van der Berg, 2008). Similarly, familial death can impact educational attainment; however, the extent of impact is variable according to the closeness of the relationship and may also be influenced by gender and race (Patterson et al., 2020). In households where death or other stressors haves negatively impacted income, young people may be discouraged from completing their education (Willis & Nagel, 2015).

As shown in findings below, education disruption can deplete student confidence, which is problematic as confidence is often an antecedent to pedagogic engagement (Cozolino, 2014; Willis, 2021). Student confidence also forms self-efficacy, which is a mediator between education opportunity and student achievement (Zysberg & Schwabsky, 2021).

In rebuilding student confidence, it is important to construct a coherent and secure narrative (Cozolino, 2014). Notwithstanding, there is an absence of narrative therapy research in conflict affected contexts (Aqtam et al., 2025a). The importance of narrative is seen in the findings below.

Disruption also depletes motivation, as students lose the energy and drive to learn due to hijacking factors. Motivation to re-engage with education requires considerable self-belief (Martin, 2002), which is often damaged during disruption by circumstances beyond the student’s control.

### Education Recovery in Southern Africa

This study was located in South Africa and included participants from South Africa and Mozambique, which is important to note as education disruptions have been frequent in these countries due to conflict and war. When COVID-19 swept the globe in the early 2020’s South Africa was already suffering institutional closures due to student protests and civil unrest (Landa et al., 2021). Further, many education policies in South Africa have roots in Apartheid (Department of Education, 2005), a time when school closures were frequent due to conflict and violence. These disruptions have had compounding effects on education outcomes in South Africa (Landa et al., 2021). The South African senior secondary matriculation rate is 57% (OECD, 2025). Due to its divided past, education outcomes have long been disparate in South Africa (van der Berg, 2008). This is compounded by high immigration rates from Mozambique and Zimbabwe (SIHMA, 2026).

Moreover, most of the country’s population (approximately 66% in 2019; Plecher, 2020) lives in rural areas, which have underdeveloped roads, lack of electricity and limited ICT access (Hlalele, 2012). As study findings show, this can mean long commutes for some students, with risks of being hijacked or mugged along the way. Gender-based violence in South Africa is the highest reported in the world, with the rate of death by intimate partners five times higher than the global average (Govender, 2023). The effects of this include sexually transmitted disease, unwanted pregnancies, mental illness and physical injuries (Govender, 2023). Study findings below demonstrate the effects of gender-based violence on education.

A portion of study participants were from Mozambique, which also has a history of political violence, war and famine (Shaw & Harris, 2003); but unlike South Africa, Mozambique’s economy and political system remain unstable. The lower secondary matriculation rate in Mozambique is approximately 32% (UNESCO, 2023). In the early 2000’s it was estimated that over half the population in Mozambique was under the age of 15 because of war (ibid.). The legacy effects of war on education outcomes, particularly for people living in rural areas in Mozambique, are still being felt (Chichango et al., 2023). There is also a noted gender disparity in economic opportunities in Mozambique (ibid.).

Given the multiplicity of disruptors, Southern African geographies present an opportunity to understand the breadth and depth of the phenomena of education disruption and recovery.

## Research Design

Phenomenography investigates and maps lived human experiences and perceptions of how learning occurs (Willis, 2018; Marton & Booth, 1997). Phenomenography is an efficacious cross-cultural methodology that has been previously used in conflict-affected settings (Willis, 2018). The suitability of phenomenography for education research is well established by Marton and Booth (1997), who assert that pedagogical opportunities are expanded when we understand the experience of an individual in their learning. The focus of investigation is the phenomenon of education recovery in and after disruption in conflict-affected settings. The intent of phenomenography is to map (-graphy) variations of lived experiences in the phenomenon (Åkerlind, 2005; Hajar, 2021; Harris, 2011) in an outcome-space. This mapping supports the development of a model and definition of education recovery, which is the aim of the present study, therefore phenomenography is more suitable than phenomenology.

### Data Collection

Semi-structured interviews (*n* = 30) were the primary data collection method. Before interviews a forerunning pilot survey (*n* = 66) was distributed online and in hard copy to ascertain preliminary perspectives of education disruption. The pilot survey was distributed in English, which is the business language of South Africa, and was open for approximately six weeks and follow up interviews were conducted intensively over seven days. This mixed methods process contributed to the trustworthiness of the study as quantitative results confirmed qualitative findings and vice versa. Survey items were fashioned from literature to investigate cognitive and affective domains of learning (Australian Curriculum, 2010; Cozolino, 2005, 2014; Marshall, 2014; Marzano & Kendall, 2006; Smith, 2021; South African Curriculum, 2021). This paper reports survey data from three sections of the survey – knowledge and skills impacted by disruption, personal attributes impacted by disruption, and supports needed for recovery. For more information about the survey design see Willis et al. (2025). Due to ethics protocols, the survey was anonymous and interview data was de-identified.

Survey items were quantitative and qualitative. Likert-style items used a three-point ordinal agreement scale, which was chosen over a five-point scale to avoid subjective interpretations of “strongly agree” and “agree” ranks and scale biases (Friedman et al., 1994) as for most participants English was a second or third language. Open ended items provided opportunity to describe incidents that aided education recovery.

Semi-structured interview questions were designed for a phenomenographic methodology with the aim of understanding education disruption and recovery experiences. Guiding interview questions included: *How was your education was disrupted? What were your experiences?* Follow up questions also included: *Did your teachers help you? If they did*,* how did they help you? What did you do to fill the gaps in your learning/education?* Questions were open-ended so that participants had the opportunity to express what was important to them.

Interviews were conducted with individuals or groups, depending on participants’ preferences, and were approximately 20 min in duration. Previous experience has shown that group interviews allow participants to outnumber the researcher and give them confidence in an interview environment. Notwithstanding, some participants preferred one-to-one interviews. One interviewee, Bonita, requested a translator to be present as she felt she could better describe her experiences in Xhosa. Her responses were in Xhosa and English as she felt comfortable and interviewer translations formed part of her transcript when she preferred Xhosa. All other interviews were conducted in English. To reduce power differences, participants were assured that the interviewer was there to learn. Interviews were audio-recorded, transcribed and de-identified.

### Research Participants

As this study investigated education recovery, not system rebuilding or short-term learning loss remediation, adult participants were chosen who could reflect on their experiences of disruption and recovery over years. They were residents of South Africa and Mozambique and had all experienced education disruptions caused by conflict during childhood and adolescence, including war and political violence, and arising poverty, sickness, political instability and crime. Snowball sampling was used for the pilot survey and interviews as the research was reliant upon the partner organisation for recruitment. This study has two participant cohorts: interview participants (*n* = 30) and pilot survey participants (*n* = 66).

Thirty adults volunteered for interviews: six men and 24 women, ranging in ages from 19 to 56 years. The gender imbalance was a product of the snowball sampling method and is acknowledged as a limitation. Interviews were conducted in person, over the phone and on video calls. Interviewees included employees of the Salvation Army, women living in shelters, social workers, childcare workers, teachers, and college students. Thirty participants constitute a robust qualitative sample size (Hennink & Kaiser, 2022).

Pilot survey participants (*n* = 66) ranged in age: 37% were aged 18 to 34 years (*n* = 11 and 13 respectively) and 33% aged 45–54 years (*n* = 22). The largest age group was 45–54-year-olds. Of the 66 survey participants, 77% were female and 23% were male.

### Data Analysis

Interviews (*n* = 30) were digitally transcribed and manually checked in multiple rounds, generating interview summaries for each participant. Finalised transcripts were manually and digitally analysed in multiple iterations using emergent thematic coding (Miles et al., 2020) to identify participant conceptions (Marton, 2004). First, a descriptive coding spreadsheet was manually generated using emergent codes and participant demographics. Second, transcriptions were coded in NVivo™ software. Emergent codes from the manual analysis were entered into NVivo™ and other emergent codes were added as transcripts were digitally re-read. Word searches ensured concepts and synonyms were captured in the coding process. To ensure trustworthiness (Miles et al., 2020), codes with fewer than three participants that could not be triangulated were either discarded or merged with other codes, unless they were sub-codes belonging to a larger code and were preserved to map the variance within the code as rare codes may represent important but uncommon experiences. Codes were tabled and heat-mapped to identify the data density of participant conceptions (see Appendix 1). Due to the phenomenographic nature of this study, codes were organised into categories of description in the table in a process of abstraction.

Due to the pilot survey sample size (*n* = 66) statistical data is considered specific to the research context and was analysed descriptively. Survey data was statistically analysed according minimum, maximum, mean, standard deviation, variance, count and percentage data. Due to the phenomenographic nature of this study, multiple crosstab analyses were conducted to investigate the relationships between survey findings and demographics, using Pearson’s *r* effect size to measure the strength of the association. Marton (2004) argued that relational thinking is needed to understand a phenomenon. Data yields were tabulated, heat-mapped and graphed, to analyse trends and patterns.

## Findings: The Phenomenon of Education Recovery after Disruption – The Long Journey

**Fig. 1:**
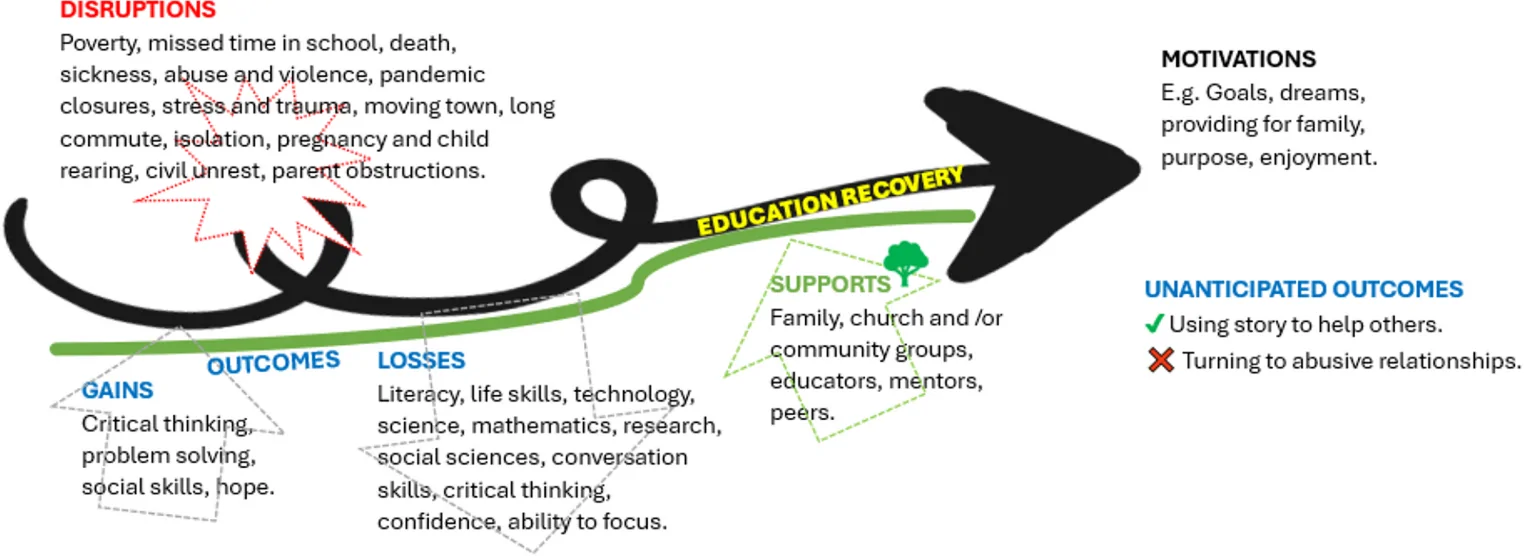
The phenomenographic outcome-space of education recovery after disruptions in conflict-affected settings: The Long Journey

Findings emerged in four major categories of description: motivations for recovery, supports for recovery, outcomes of disruptions, and causes of disruptions, which are explained in the sections below. Phenomenographic data presentation brings together categories of description in a modelled outcome-space to graphically depict the phenomenon (Åkerlind, 2005; Hajar, 2021; Harris, 2011). This study’s graphic outcome space is entitled *The Long Journey* (Fig. 1). *The Long Journey* illustrates the complex nature of education recovery, providing insights into what can be done in local contexts to support recovery efforts. Due to the large number of codes in each category of description, only indicative data samples are provided below.

### The Phenomenon Explained: Motivations for Education Recovery in Conflict-Affected Settings

A key category of description in the phenomenon of education recovery after disruption in childhood and adolescence is the driving motivations that re-engage disruption-affected people in education into their adult years. Interview data was illuminating in this regard. Participants discussed future goals, the need to provide for family, and a sense of higher purpose.

#### Goals and Dreams

Evidently, goal setting acts as a mediator between disruption and recovery. Participants were driven or “pushed” by goals, dreams and hopes (14 participants, 40 references).

*Mary: I said to myself*,* I want to be somebody*,* I want to become somebody in this world. I said I [can’t lead the] world by just being a cleaner… you know you want to earn a better salary.*

*Maxi: I just want to have that matriculation. Because the life without matriculation is not good … my goal actually pushed me.*

Survey data also showed goals motivated individuals to recover learning. Goal setting in education recovery contexts may be driven by a need to take back control (Martin, 2002). This requires significant resilience as recovery students may not be able to control sabotaging factors like poor health.

To realise these goals and dreams, participants needed support. These participants were actively recovering their education, but all were also receiving support from families, employers or the Salvation Army. Goals and dreams need support when personal resources come short.

#### Providing for Family

Although family responsibilities disrupted education for many young people, they also pushed them back to education. Seven interview participants (11 references) mentioned the driving need to provide for their families. This sense of responsibility propelled them forward.

*Geraldine: I need to get my act together*,* for my sake and my son’s sake.*

*Mary: …having a child … I had to go back to school.*

Qualitative data from the pilot survey affirms this finding:

…a poverty life not being able to take care of myself and my child… it really motivated me to go back and finish my studies.

Participants were both pushed by fear of poverty and pulled by hopes for a better future for their children.

#### Higher Purpose

A sense of higher purpose also came forward in the qualitative data. Four interviewees (seven references) directly spoke of this impression. For example:

*I was in college*,* doing the welding things*,* I had no love*,* I was just keeping time*,* because I had nothing to do. Yeah*,* so then it’s where I decided it was okay. Let me go where my heart is leading me to. Maxi*.

As one survey participant stated, *“realising who I am and what I do is for my development and future impact onto my society”* conveys a sense of purpose. Purpose helps individuals remain motivated and focused on their goals (Martin, 2002). Education was a means to recovering a sense of purpose after disruption.

### The Phenomenon Explained: Supports for Education Recovery in Conflict-Affected Settings

A key feature of the phenomenon (Fig. 1) supports that undergird recovery. Both the interview and pilot survey datasets contribute to this category of description as they explicate meaningful relationships that braced education recovery. Interview participants acknowledged the support of the Salvation Army (11 participants, 17 references), teachers (10 participants, 10 references), employers (nine participants, 17 references), and families (six participants, 16 references) (Appendix 1). Supports described were pedagogical (e.g. study help), financial, physical (e.g. shelter and food), and moral.

In the pilot survey, participants were asked to identify who encouraged them to grow and learn. Parents and church leaders were the two highest scoring “encouragers,” with 30% and 22.2% respectively. Crosstab analyses showed approximately 50% of all age groups, except 25–34-year-olds (15.4%), acknowledged parent support. Church support was most acknowledged (83%) by 55–64-year-olds. Thereafter, participants nominated other family members (13%), other important people (10%), older siblings (8%) and friends (7%) as supportive. Where family capacity was insufficient, participants relied upon community supports, like the Salvation Army, and systemic supports like government funding, for education recovery. Community and government support is vital for equitable education recovery opportunities.

### The Phenomenon Explained: Outcomes of Education Disruptions in Conflict-Affected Settings

As Fig. 1 shows, disruptions caused gaps, losses and gains in knowledge, skills, and personal attributes in childhood and adolescence. This category of description includes two unanticipated outcomes: leveraging disruption stories to help others and turning to abusive relationships for survival in conflict-affected settings.

#### Knowledge and Skills Gaps and Losses: Qualitative Findings

Eleven interview participants talked about knowledge and skills gaps and losses (19 references). Upon return to study after disruptions during adolescence, Sally felt unprepared and distanced from education. Her challenge was to close the psychological gap between herself and her studies, and fill gaps of missing information:

*It was not easy to catch up information*,* and I’d understand what I needed to do and sometimes*,* like my mind was not prepared to have information in it. It was distance from what I wanted.*

This highlights the importance of how education gaps are conceptualised. Evidently, gaps in knowledge and skills adversely affect esteem.

##### Illiteracy

Within this sub-code, three participants specifically discussed the impacts of illiteracy.

As an example, Tamia dropped out of high school to have a baby and laments gaps in her literacy, which she described as a “heavy burden”:

*I can’t read properly. … I never had experience reading one of that books [sic] …And my heart feels so sore because I wanted that experience … And my spelling also… because what if my child might become one day to me just to help him with his homework? And I don’t have a clue that’s going on.*

Tamia’s comment demonstrates the intergenerational transmission of education disadvantage – a vexatious issue that is discussed further below.

##### Life Skills

Two participants spoke directly about the impacts of education disruption on their life skills. Alecia did not know to wear underwear until she was 13 years old. Nor did she know about menstruation or contraception. She felt stressed because she was “ruling” her life without adult help. This lack of knowledge decimated her esteem.

##### Negative Impacts on Knowledge and Skills: Quantitative Results

In the pilot survey, participants rated the negative impacts of disruptions. In the “very negatively impacted” rank, public speaking (39%, SD1.01) was the highest, followed by science and mathematics knowledge and skills (34% respectively, SD 0.9 and 0.83). The highest ranked “not negatively impacted” skill was writing (43%, SD 0.85).

When combining “very negatively impacted” and “negatively impacted” categories, science (76%, SD 0.83), mathematics (68%, SD 0.9) and research skills (67%, SD 0.93) ranked as most negatively impacted (Table 1). The vulnerability of science, mathematics and research skills is not surprising, as these disciplines require sequential and scaffolded learning. This is discussed further below.

**Table 1:** Negative impacts of education disruption on knowledge and skills (combined ratings from survey data *n* = 66)

Knowledge and Skills	% of negative impact	*N*	SD
Science	75.86%	44	0.83
Mathematics	67.80%	40	0.9
Research - collecting and analysing information	67.28%	37	0.93
Social sciences (history, geography, law and politics)	63.64%	35	0.85
Personal management (e.g. getting your work done on time)	62.71%	37	1.06
Participating in conversations	61.01%	36	1.03
Critical thinking (making judgements and evaluating)	58.62%	34	1.08
Reading	58.33%	35	0.97
Technology	56.14%	32	1.05
Public speaking	55.93%	33	1.01
Social skills - ability to relate to and interact with others	55.93%	33	1.11
Creativity	54.24%	32	1.01
Problem solving	53.44%	31	1.05
Writing	49.15%	29	0.85

Survey data is descriptive and specific to the research context

Above 60% of survey participants rated social sciences (64%), personal management (63%) and participating in conversations (61%) negatively. No significant relationships between knowledge and skills gaps, cause of disruption, or length of time of disruption were found in the survey data.

The age groups reporting the most negative disruption to mathematics and science knowledge and skills were 25–34 year olds (*n* = 13; 55% mathematics and 53% science) and 45–54 year olds (*n* = 19; 60% mathematics, 52% science).

Men reported higher negative impacts on problem solving, social skills and critical thinking (69.2% respectively) than women. Conversely, women reported higher rates of positive outcomes in the same areas.

#### Knowledge and Skills Gains

The pilot survey also showed positive outcomes of disruption. The most highly rated skills gains were critical thinking (22%, SD 1.08), problem solving (21%, SD1.05), social skills and personal management (18%, SD 1.11), participating in conversations (15%, 1.03), and creativity (15%, SD 1.01). Overall, women reported more positive outcomes than men.

#### Personal Attributes Affected by Disruption

Disruptions impacted personal attributes. The pilot survey investigated confidence, resilience, ability to focus, optimism and self-efficacy, showing confidence as the most negatively affected personal attribute (52% agree, SD 0.88). Interview data confirms this result as is presented below. The next most negatively affected personal attribute was “ability to focus and self-regulate” (49% agree, SD 0.86), followed by optimism (47% agree, SD 0.81), self-efficacy (44% agree, SD 0.89), and resilience (41% agree, SD 0.81).

Males rate higher in all negative ratings than females, except optimism, which is similar. Over 60% of male respondents reported negative impacts on confidence, resilience, ability to focus and self-regulate, and self-efficacy (beliefs about abilities). Less than half of the female respondents reported negative impacts on self-attributes.

A crosstab analysis of personal attributes by age revealed that 18–24-year-olds were most affected (Table 2). The vulnerability of young adults cannot be overlooked in these results, reporting highest negative impacts on confidence (72.7%), focus (72.7%), and self-efficacy (81.8%). The other age group of concern is 25–34-year-olds and their negative ratings of resilience (53.8%). Amongst 35–44-year-olds, half report negative impacts on confidence, ability to focus and self-regulate, and optimism.

**Table 2:** Negative impacts of education disruption on personal attributes by age (survey data *n* = 66)

		18–24 years old	25–34 years old	35–44 years old	45–54 years old	55–64 years old
Total Count (Answering)		11.0	13.0	10.0	19.0	6.0
Confidence	Agree	72.7%	46.2%	50.0%	47.4%	50.0%
	Neither	27.3%	30.8%	10.0%	5.3%	16.7%
	Disagree	0.0%	23.1%	40.0%	47.4%	33.3%
Resilience	Agree	54.5%	53.8%	40.0%	27.8%	40.0%
	Neither	36.4%	38.5%	20.0%	33.3%	20.0%
	Disagree	9.1%	7.7%	40.0%	38.9%	40.0%
Ability to focus and self-regulate	Agree	72.7%	30.8%	50.0%	47.4%	40.0%
	Neither	9.1%	38.5%	20.0%	21.1%	20.0%
	Disagree	18.2%	30.8%	30.0%	31.6%	40.0%
Optimism	Agree	54.5%	38.5%	50.0%	47.4%	50.0%
	Neither	27.3%	38.5%	20.0%	31.6%	0.0%
	Disagree	18.2%	23.1%	30.0%	21.1%	50.0%
My beliefs in my own abilities	Agree	81.8%	38.5%	30.0%	47.4%	0.0%
	Neither	9.1%	15.4%	40.0%	5.3%	60.0%
	Disagree	9.1%	46.2%	30.0%	47.4%	40.0%

Survey data is descriptive and specific to the research context

##### Confidence Losses

Six interview participants described confidence losses arising from disruptions to education. The qualitative dataset shows that a loss of confidence affects esteem and identity.

Patti explained how disadvantage affected confidence:

*I have to overcome the mindset of inferiority…. And the mindset of if you come from a very disadvantaged family*,* nothing good will ever come from that family.*

As confidence is an antecedent to education outcomes (Willis, 2021); it is vital that educators find ways to rebuild students’ esteem and efficacy. This requires strong teacher-student relationships and intentional teaching strategies.

##### Hope Gains

Five interview participants (10 references) indicated they had gained hope for the future. For example, Bonita described a future of working and independent living. Living in a women’s shelter, she had a plan to rebuild her life after complex traumas, including brain injury, hoping for a peaceful future. Bonita enjoyed cleaning and exercise and found those two activities helped settle her mind. She was saving money from a disability grant and hoped to use it for housing. Her resilience and hope were palpable.

There are intersections in the interview dataset between *hope* and *leveraging story to help others*. Participants who found hope wanted to encourage others in similar situations. There is a reciprocity between hope for the future and telling your story that could be leveraged in recovery efforts.

#### An Unanticipated Outcome: Leveraging Story to Help Others

Six participants described using their story to help others as a positive outcome of their experiences. For example, Sally experienced considerable disruptions to her education over decades, not matriculating high school until well into her forties. Her determination and resilience were evident, and she wanted to encourage others who had also experienced hardships.

*We may cry when it comes*,* but it is a way of preparing us for what is to come. We are learning from hard experiences…It is hard sometimes*,* but we have to pass through deserts. These deserts will help us to become better people.*

Patti also used her story to encourage others, but she was careful how she told it so not to re-traumatise people. She wanted to convey value on the next generation as she did not feel valued as a young person.

*… stones can be thrown at you. But you can also use those stones to build a house… I was not seen*,* but I will make sure … that our young people of the next generation are valued.*

This finding evidences the power of story to give students hope for their present situations. The characters in the stories represent possibility. Stories pull lessons from the past for the sake of the present and future. The value of community and story in rehabilitation is established (Willis et al., 2021) but these findings affirm the importance of mentoring and narrative (Cozolino, 2013) in education recovery settings, and the utility of narrative therapy in conflict affected settings (Aqtam et al. 2025a).

#### A Tragic Outcome: Turning to Abusive Relationships and Prostitution

One of the tragic outcomes of education disruption was the perpetuation of abuse and prostitution. This sub-code comprised five participants and nine references. Personal data excerpts are not provided here to protect participants. However, Diedre and Nora (social worker and teacher) lamented working with adolescent girls who abandoned their education for abusive relationships:

*Diedre: They left education the minute they leave here. And then they gave themselves to men and men are abusing them… You don’t have education*,* you don’t have any skill … Everything that you that you need to survive… So it really pains me to see them…*.

*Nora: They’ve got that mindset that for you to have something you must have a man.*

*Diedre: Yet they see the very men who are doing things for them are the very ones that are taking their lives. South Africa*,* we have the highest statistics of women that die at the hands of men*,* compared to the entire world. We tell them that every single day… They do not even hear what we are saying.*

This is a very vexatious finding. Evidently, socio-cultural pressures and the associated pressures of poverty hijack education. This code intersects with abuse, violence, stress and trauma in the qualitative dataset and confirms the prevalence of gender-based violence in Southern Africa (Govender, 2023, UNGEI, 2024). Although very complex and difficult to address, it cannot be ignored.

### The Phenomenon Explained: Causes of Education Disruptions in Conflict-Affected Settings

**Fig. 2:**
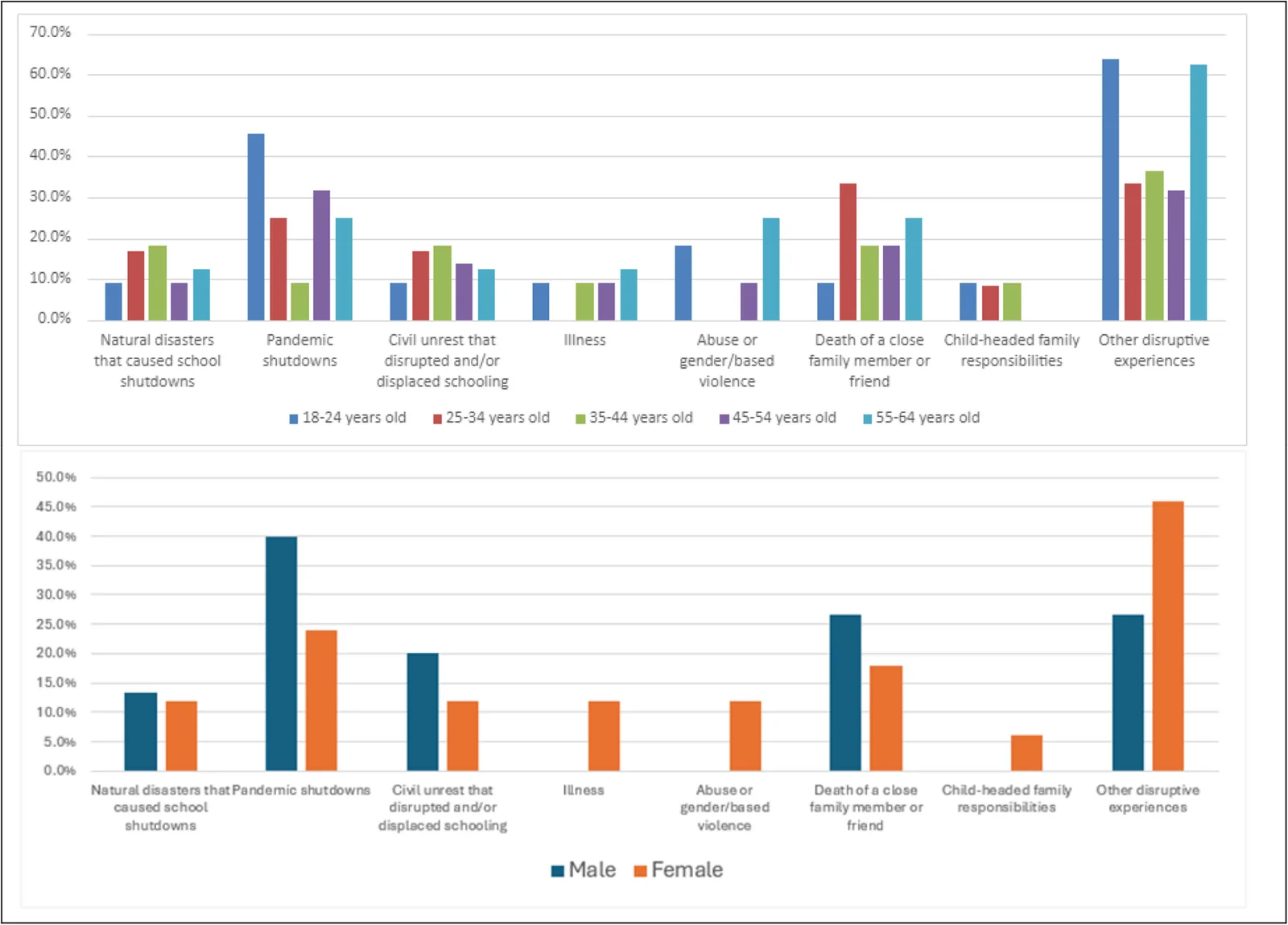
Causes of education disruption by age and gender of survey participants (*n*=66). Note: Survey data is descriptive and specific to the research context. Males *n*=15. Females *n*=50. 18-24 years *n*=11. 25-34 years *n*=12. 35-44 years *n*=11. 45-54 years *n*=22. 55-64 years *n*=8

**Fig. 3:**
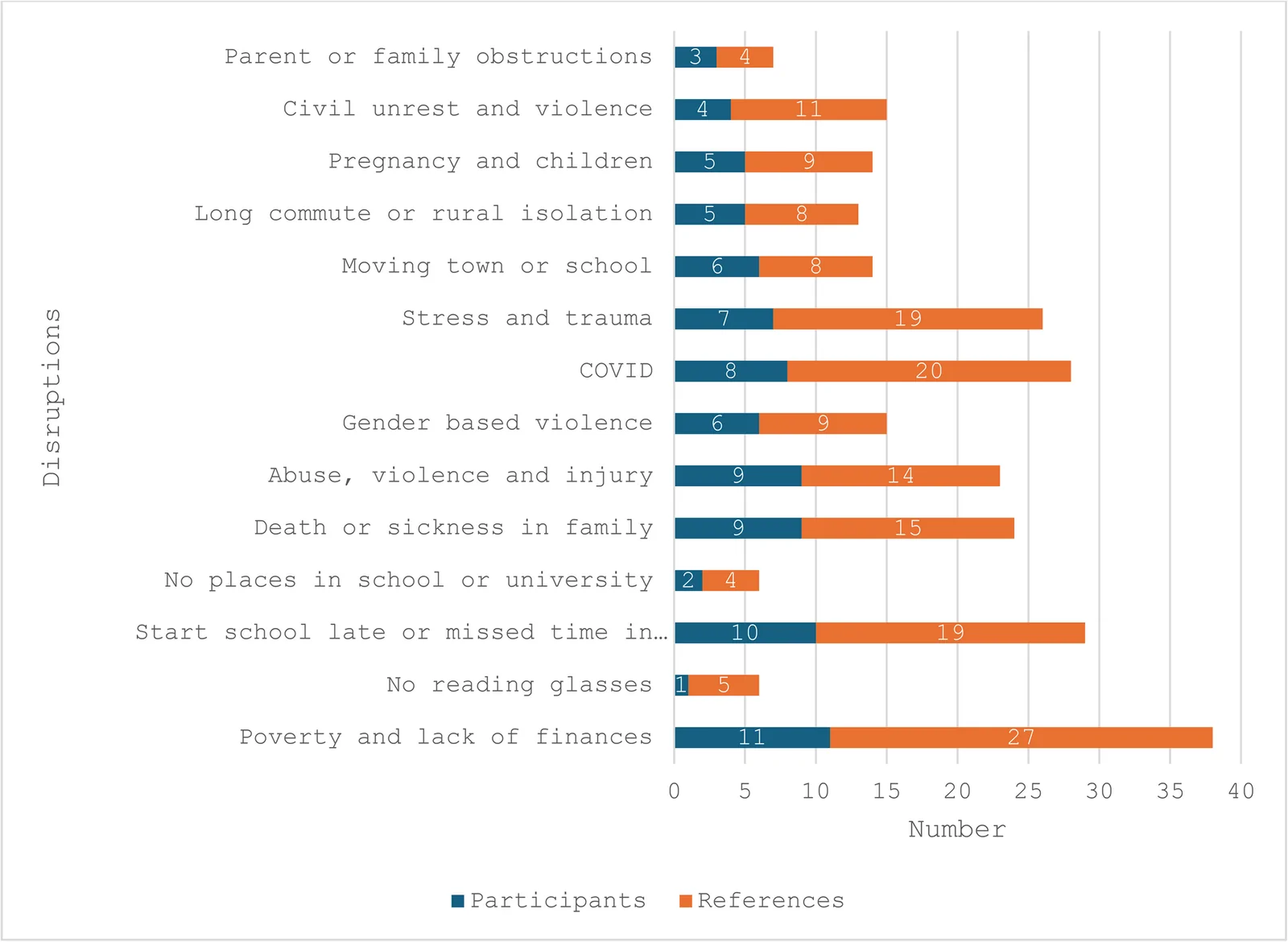
Causes of education disruption: Codes and sub-codes (interview data *n* = 30)

Viewing survey results and interview findings together, poverty, sickness and death (including COVID-19), and violence (civil and domestic) emerge as root issues of education disruption (seen Figs. 2 and 3 below) in conflict-affected settings. These disruptions caused uncertainty and subsequently threatened health and wellbeing. As discussed below, there is a need for decision makers to triage causes of disruption so that root causes are being addressed as a priority.

Within this category of description, financial hardships were the most common cause of education disruption in the interview data and pilot survey data correlated poverty with depleted confidence and self-esteem. Violence related causes of disruption also stand out in the datasets, including abuse, gender-based violence and civil unrest.

Education was often not recovered in the season it was lost. For example, death of a close family member or friend could have ramifications for five or more years, and implications of having to move town because of sexual violence or civil unrest in childhood could permanently change one’s life trajectory. Figure 2 outlines main causes of disruptions in conflict-affected settings identified in the survey by age and gender of participants.

Phenomenological analysis of the interview dataset (*n* = 30) yielded 14 codes and sub-codes in the causes of disruptions category (Fig. 3). Many of these codes align with the survey results above; however, the qualitative dataset shows more variation in the phenomenon.

**Fig. 4:**
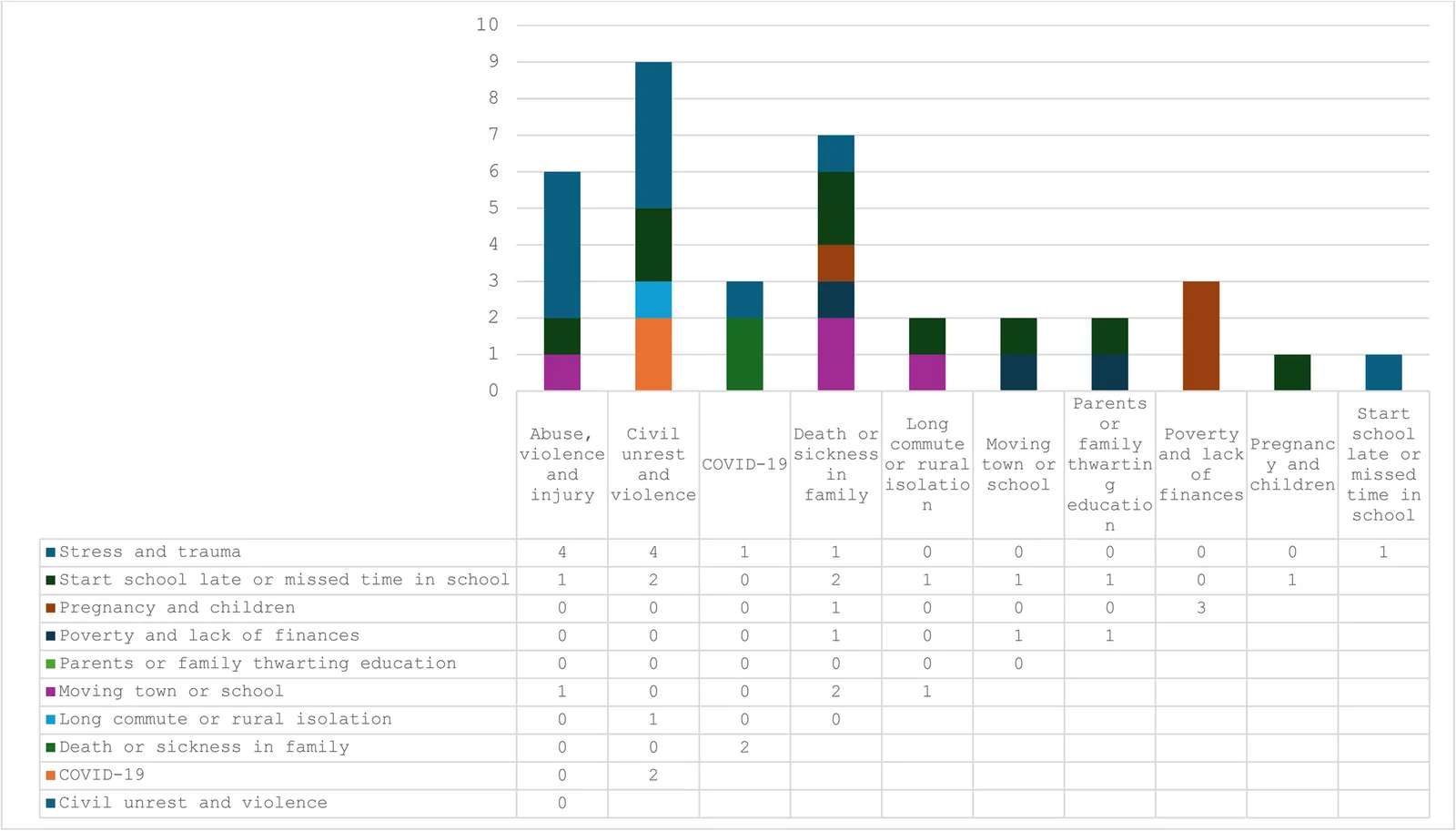
Intersections between causes of education disruption codes in the interview dataset (*n* = 30)

There were noticeable intersections between the causes of disruption in the qualitative interview dataset (Fig. 4). Notably, the *stress and trauma* code intersected with six other codes.

Causes of disruption data sections below are ordered by coding density, with most common causes presented first.

#### Poverty and Lack of Finances in Conflict-affected Settings

The most common cause of education disruption in the interview dataset was poverty or financial difficulty during childhood. Eleven interviewees (27 references) described how a lack of finances thwarted their education. Some participants went to work instead of school when they were children. Many dropped studies because they could not afford living expenses, pay tuition fees, travel, or afford study materials. Housing insecurity also disrupted education. Many found labouring jobs, but many could not work because of injuries or pregnancies (see intersections in Fig. 4). Multiple interviewees described the disadvantage of generational poverty. Here is an example.

Self-described as “the mother of myself” Alecia did not have enough money for school clothes:

*it was very hard for me because I’m not the same as other children in school …because I never buy a uniform. I never wear any shoes for school. I never wear socks… It was not nice for me because no one was gonna do it for me.*

At the time of the interview, Alecia was living in a women’s shelter and recovering from years of violence and poverty. Her story is woven throughout the findings below.

Poverty could, in part, be systemically addressed, although the cultural effects of poverty are complex (van der Berg, 2008). Interview findings and literature both show that poverty exacerbates the effects of other disruptions (e.g. Maly & Sakurai, 2023; Srivastava et al., 2021), which in turn compound education disruption. Although education is often seen as a solution to poverty, paradoxically, it is also undermined by poverty.

#### Start School Late or Missed Time in School in Conflict-affected Settings

The second most common reason for education disruption in the interview dataset was missed time in school (10 interviewees,19 references). Some participants started school late, while others dropped out due to sickness or civil unrest. This code intersects with most other codes (see Fig. 4).

As an example, Kia’s family was not educated so schooling was not prioritised and generational disadvantage perpetuated. All children began school at the same time, meaning Kia began school six years late:

*The family was not affording because no one was educated… We started all in the same time going to school. I was 12 years when I started school.*

Kia was 54 years old at the time of the interview and had experienced multiple disruptions - lack of parental education, geographical isolation and violence all contributed to missed time in school. This could, in part, be systemically addressed through policy or parent education; however, a low value for school is a socio-cultural issue that requires a long-term commitment to cultural change.

#### Death or Sickness in the Family in Conflict-affected Settings

Nine participants (15 references) attributed familial death or sickness to dropping out of school. Some participants became child or adolescent heads of their households:

*I was struggling a little bit*,* but I did go well until she died [in] Grade 6. She told me*,* “oh Alecia I’m sick now… so you gonna be alone. So will have to look after yourself.” … I try to go to school and then after that she passed on. Then I have to go by the street and stay there… I was a mother of myself.*

Alecia never finished school. Similarly, Geraldine went to work when her mother got sick and never matriculated. When Sove’s mother was hospitalized, she had no one at home to make her go to school. Mercy had to move schools because of her father’s sickness; and subsequent hunger and poverty. Death or sickness during childhood are root causes for education disruption, but it is the arising financial difficulties, forced relocations or family responsibilities that keep students out of school (see Figs. 3 and 4). This again highlights the need for comprehensive solutions and decision makers to address root causes of disruption.

#### Abuse, Violence and Injury in Conflict-affected Settings

Nine interview participants (14 references) discussed the effects of abuse, violence and injury, which is identified as a root cause of disruption in this study. Alecia’s story is extended here as an example. She had a violent start to life, abandoned as a baby with head injuries, she was raised in poverty and her schooling was delayed.

*There was a fight between my mother and my father… my father beat me up on my head … so they think I’m dead. And then after that they run away… They gonna bury me in the morning. Me*,* 5 o’clock in the morning*,* I wake up… the stepmother took me… but she was suffering with food [scarcity]*,* so she couldn’t take me to the hospital… she didn’t have money*,* so she have to go to the mountain and take the leaves*,* try to heal my head. But it didn’t heal right… from three months until I was 10 years struggling… Then after that I have to start late to the school.*

This code recurrently intersects with the stress and trauma code (Fig. 4), again demonstrating the onflow effects of abuse or violence. Stress and trauma might hijack learning and education (Willis & Nagel, 2015), but the root causes of violence and abuse must be addressed to instigate positive change.

##### Gender-based Violence

Six interview participants (nine references) discussed the effects of gender-based violence on their education. For many, violence hijacked their focus and attention in school, for some they had to physically heal before they could resume their studies. Sally stopped attending school because of bandits who preyed upon girls on the road to school.

*…on that the same part of the river people were hiding themselves there*,* so that any girl who passes alone they can grab and rape. So it was very hard for us [girls] to go through that experience. So I had to… just sit at home*,* and I couldn’t go anywhere because of those things.*

Sally also explained schoolteachers were sexually abusive toward female students, confirming the atrocity of school-related gender-based violence in Southern African countries (UNGEI, 2024). Sally is now in her late forties and recently matriculated high school after many disruptions.

Jerry explained that school was a distraction from worrying about his mother:

*when you’re at school*,* you’re in a good place*,* you forget about the problems that you have. But when the bells at half past three started to ring you started to think about what is going on at home… you think about what’s happened to my mum*,* what’s if she’s dead?*

At the time of the interview, Jerry had completed Year 10 and was enrolled in vocational education.

Due to the high rates of gender-based violence in South Africa (Governder, 2023) and Mozambique (UNGEI, 2024), this vexatious and complex issue warrants comprehensive responses. Evidently, gender-based violence perpetuates cycles of low education and poverty.

#### Global Pandemic in Conflict-affected Settings

The effects of COVID-19 were widely felt amongst the pilot survey (20%) and interview participant groups. Eight interview participants (20 references) explained how the pandemic thwarted their education. This code intersects with the *death and sickness* code, which in turn intersects with *stress and trauma*,* staring school late*,* poverty* and *moving town or school* codes (Fig. 4). The effects of COVID-19 are far reaching and will continue to impact education outcomes for years to come (Engzell et al., 2021; Psacharopoulos et al., 2021).

#### Stress and Trauma in Conflict-affected Settings

The stress and trauma code has multiple intersections with other causal codes, notably with *abuse*,* violence and injury* and *civil unrest.* Seven interview participants (19 references) mentioned the impacts of stress and trauma on their education. Trauma is intrusive, hijacking attention and emotional resources (Aqtam et al. 2025c). Many of them were grateful to the Salvation Army for time and space for trauma recovery.

As described above, Alecia suffered considerable child abuse and subsequently experienced the effects of stress and trauma:

*So I was struggling even there at school to understand what teacher is teaching… because* ***my past was following me*** *…So I sit there [at school] …when I look at the children*,* when I see they are very happy and then me I still have a question mark about my father and my mother? Why they did this to me and then they ran away then that was a pain for me. … I was thinking like I’m not a human being. Why? That’s why I was always crying. …It was very painful for me …Because I go through a lot for my life and* ***I was fighting for education***. *I was fighting for me to get a better place.*

Stress and trauma hijack learning (Aqtam 2025; Aqtam et al. 2025a; Willis et al., 2021) and add an emotional neurobiological load to education experiences (Cozolino, 2005).

#### Moving Town or School in Conflict-affected Settings

Six interview participants explained how moving towns or schools interrupted their education during childhood. For some it was because their parents moved for work, for others it was to escape abuse, or due to family death or sickness. This code is difficult to address as it is both a symptom and cause of education disruption. Accordingly, causes of disruption ought to be triaged.

#### Long Commute or Rural Isolation in Conflict-affected Settings

Five interview participants (eight mentions) discussed the impacts of long commutes or rural isolation on their education. McGates did not have electricity or running water in his rural home when he was a child. He had to walk a long way to school and spent time drawing water and doing chores when at home. Education is often thwarted by basic survival needs in rural contexts in many nations, including South Africa (Hlalele, 2012; Plecher, 2020).

#### Pregnancy and Children in Conflict-affected Settings

Five interview participants (nine references) explained that pregnancies and parenting responsibilities ended their secondary or tertiary education. Importantly, there is a salient need to support adolescent mothers to recover their education after child rearing, as Tamia’s remarks demonstrate:

*I fell pregnant in Grade 9 and I thought I could complete school. …but I gave birth at 34 weeks to a premature baby*,* and then I had no choice to drop out to take care of my baby. Tamia*.

The pregnancy and child rearing code intersects with the *poverty* code in the interview dataset, demonstrating the subsequent effects of pregnancies and child rearing for women. Family and organisational supports are vital for supporting women’s education, but systemic support is needed to alleviate strain on families.

#### Civil Unrest and Violence in Conflict-affected Settings

Interview participants (*n* = 4; 11 references) described occasions where civil unrest made it impossible to continue education. Sally had to move away from the city due to war, and her schooling ceased soon after. Francis experienced civil unrest during the Fees Must Fall Movement, and she explained that ways of learning were never the same thereafter. Mercy’s education was broken because of Apartheid violence, and Fiki’s matriculation was delayed.

*I remember when… teacher was busy teaching*,* guns*,* you know*,* and we had to go out… I remember I fell and my knee could not work properly and we had to run to our homes and some got injured*,* heavily. It wasn’t nice. My matriculation was even impacted by that. I had to supplement some of the subjects the following year. So my matriculation is two years instead of one year. …in the midst of the night you’re still sleeping*, guns *click*. Fiki.

Political violence disrupts education and places an emotional burden on students (Aqtam et al. 2025b). This present study demonstrates the long-term effects of conflict on education recovery.

#### Parents or Family Obstructing Education in Conflict-affected Settings

For three interview participants, education was obstructed by a lack of family support during childhood. Bonita’s mother refused to sign a bursary application form. Consequently, she could not afford to travel to school so dropped out. Victoria’s uncle often bought school supplies for her, but his wife took them. Joseph’s father had five wives and he was one of 29 children. Being the youngest, his older brothers should have assisted school expenses, but this never eventuated.

Children cannot support themselves through school; they rely on their families. If the family does not value education and support them, then generational disadvantage is perpetuated.

## Discussion: A Definition of Education Recovery after Disruption in Conflict-affected Settings

In response to the research questions and drawing from the phenomenographic model (Fig. 1), this study provides a first iteration of a definition of education recovery after disruption in conflict-affected settings.

Education recovery after disruption is a long-term, future-focused journey of filling gaps, progressing learning and regaining confidence in education, enabled by proximity to opportunity and supports. Education disruption is a social problem that distances people in conflict-affected settings from education engagement. Education disruption creates gaps between the person and the experience of education, causes uncertainty and threatens wellbeing. Young people are particularly vulnerable to its negative impacts. Gaps might first form because of poverty, violence or ill-health, for example, but are widened by ensuing complications such as trauma, illiteracy or low confidence. For this reason, causes of disruption need to be triaged so that root causes can be systemically addressed to enable recovery. Education recovery is distinct from system rebuilding and short-term learning loss remediation. It ought to be viewed circumspectly and comprehensively; that is, the breadth and depth of recovery experiences and strategies may have cultural, contextual and gendered nuances.

### Practical Implications

Some disruptions are specific to geography; others could happen anywhere. Some knowledges and skills (e.g. mathematics and science) are more vulnerable to the negative effects of disruption due to their reliance on sequential and scaffolded learning. Some disruptions are episodic and ramifications are not enduring; other disruptions have permanent consequences. For some people, recovering education opportunities is possible. For others, recovery goals are more concerned with sustainably managing the ongoing consequences of disruption (e.g. physical injury from abuse). Policy makers ought to take a holistic approach to recovery and implement mandatory trauma-responsive teacher training. This may also include providing educators with resources to assess the effects of disruption – cognitive and emotional (Aqtam, 2025) – and implement initiatives for recovery.

#### Identifying and Triaging Root Causes of Disruption

Findings point to the need for decision makers to triage causes of disruption so that root causes are addressed systemically. In this study, root causes of disruptions to education in conflict-affected settings were poverty, ill-health (sickness and death, including COVID-19), and violence (civil and domestic). However, it was often the subsequent financial difficulties, emotional burden, forced relocations, or family responsibilities that kept people out of school or further study. For example, providing housing and childcare services for women who have suffered gender-based violence may enable them to recover their education and reduce the likelihood of returning to abusive relationship as a means of survival.

There is an evident reciprocal relationship between infrastructure security (housing, water, electricity) and education recovery in conflict-affected settings. Infrastructure is the responsibility of government; whether by enabling private investment or by direct provision.

In this context it behoves policy makers to address poverty, health and violence as a priority. This is a long game and will require political fortitude, but without addressing root causes governments will spend copious amounts of money on symptoms and fail to meet SDG4. Policy responses may include incentives for school attendance to discourage adolescent labour, mobile health clinics, and mental-health programs that address emotional regulation, aggression and violence.

Young women are particularly vulnerable to gender-based violence, pregnancies and ensuing health issues. Bi-partisan, multi-election-cycle commitments to ending violence against women are urgent. Policy responses should include pregnancy and childcare support for adolescent and young women and violence intervention programs. Young men are also vulnerable to negative impacts on confidence, resilience, ability to focus and self-regulate. Recovery programs may need to be gender specific so that the needs of men and women are respectively and culturally addressed as trauma can be gender nuanced (Aqtam et al. 2025c).

#### The Critical Window of Young Adulthood in Education Recovery

The vulnerability of young adults cannot be overlooked in these findings. Data show young adults are often without reference points for healthy living and learning in conflict-affected settings (Willis & Nagel, 2015) due to a lack of mentors and infrastructure securities (e.g. housing, schooling). The need for targeted interventions to boost the confidence, focus and efficacy of young people is apparent. Policy makers need to prioritise support for matriculation, in some instances starting with primary school attendance.

#### Support for Sustained Motivation

People frequently bring themselves back within proximity of education opportunities when there are pressing needs (Feuerstein et al., 2010) – for example providing for a family or attaining gainful employment. However, these needs must convert to sustained motivation to override the hijacking forces that previously distanced them from education opportunities and supports. Where need does not covert to motivation, or the hijacking stressors are overwhelming (Willis & Nagel, 2015), then community leaders need to identify ways to bring education supports and opportunities close to people who have suffered education disruptions so that recovery can commence informally. Motivation is underpinned by healthy self-belief (Martin, 2002), which may need to be built before formal education recovery can begin. The journey from disruption to healthy recovery requires systemic (education and welfare) and community support so that a degree of circumstantial control can be returned to the student. In the interim, finding ways for education to be uplifted by virtue of proximity to mentors, stories of resilience (Cozolino, 2014; Martin, 2002) and informal learning opportunities to fill confidence gaps and decrease stressors is a priority. In that way, individuals might be brought closer to formal education opportunities in the future, thereby breaking dysfunctional cycles of abuse.

### International Comparison

Findings in this study concord with similar studies in Uganda, Ethiopia and Palestine. That is, students are traumatised by conflict (Aqtam et al., 2025b) and their academic outcomes suffer accordingly (Gebre-eyesus & Reda, 2025). There is a need for gendered analyses in conflict-affected settings (Aqtam, 2025) as trauma responses can be nuanced and recovery initiatives ought to be culturally grounded (Aqtam, 2025). Community support is vital to recovery (Willis & Nagel, 2015), especially when personal resources are exhausted and encouragement is needed to push past the inertia of fear or fatigue.

### Study Limitations and Further Research Opportunities

There is a present research opportunity to test this phenomenographic model in other contexts to address causes of disruption to enable recovery. One of the limitations of the study was that participants were receiving support from the Salvation Army. Findings may not represent disruption-affected people who are not connected to formal support services. There is an opportunity to conduct research with people who are not receiving support.

It is also acknowledged that retrospective recall gives an adult perspective on childhood experiences. These perspectives are valued but cannot be considered child perspectives. Memory may be affected by time and experience. For participants where English was a second or third language, communication may lack nuance. Further, males are under-represented in the present study due to snowball sampling recruitment and results may reflect gendered experiences. An opportunity exists to better understand male perspectives.

Another limitation is the 3-point Likert scale to reduce subjective interpretations for second language speakers, a 5-point scale may yield more granular results. The sample size (*n* = 66) precluded inferential statistical testing; findings are descriptive, context-specific and not generalizable. Survey items were not validated in this population.

The use of story to help others recover was a heartening finding. There is an opportunity to further investigate the efficacy of storytelling in education recovery in conflict affected settings as there is a known absence of narrative therapy in these settings (e.g. Aqtam et al. 2025a ).

## Conclusions

This study provides the first empirically grounded definition of education recovery after disruption in conflict-affected settings, which was derived from the phenomenographic model (Fig. 1). Evidently, recovery is future-focused, enabled by proximity to supports, and critically dependent on addressing root causes of disruption: poverty, violence, and ill-health. For young adults (the most vulnerable group) gender-specific, trauma-responsive interventions are urgently needed. Policy makers should prioritise: (1) infrastructure security (housing, water, electricity) as a prerequisite for education recovery; (2) incentives for adolescent and young adult mothers to support education recovery; (3) mandatory trauma-responsive teacher training; and (4) bi-partisan, multi-election-cycle commitments to addressing gender-based violence. Future research should test these findings in other conflict-affected settings and evaluate the efficacy of specific pedagogical strategies (Willis et al., 2025) for closing learning gaps.

These findings have meaningful implications for social services so that informal and formal education opportunities are brought close to people needing to recovery their education. The phenomenon of education recovery after disruption model (Fig. 1) makes salient the criticality of organised support in education recovery efforts in conflict-affected communities. Beyond implementing programs and rebuilding schools, this research demonstrates the importance of social groups for recovery, including families, mentors, community groups, and community education initiatives. Notwithstanding, these findings also have meaningful pedagogical implications as wrap-around supports are needed for conflict- and disruption-affected students (Willis et al., 2025).

As a priority, governments need to address infrastructure – housing, electricity, water – so that young people have the best chance of education recovery. When basic needs such as housing (Maly & Sakurai, 2023) and food security are taken care of, people can turn their attention to social needs like education, employment and looking after family. This provides an alternative to re/turning to abusive relationships. Organisations like the Salvation Army create spaces where disruption-affected people can press pause on their chaos and stress and focus on recovery.

Now that the phenomenon of education recovery after disruption has been modelled and defined, research efforts can turn to efficacious methods for closing gaps for conflict-affected students, giving priority to addressing root causes of disruption. This may not yield quick political gains, but the criticality of addressing root causes cannot be overstated. Bi-partisan approaches that extend beyond a political election cycle are needed to shift the dial on such vexatious issues.

Findings from this research study ought to be tested in other contexts to map the phenomenon of education recovery after disruption across different conflict-affected settings. Indeed, disruptions occur in all societies. Opportunities for recovery, however, are not equal. This is an area for future investigation so that local decision makers are furnished with adequate research-based evidence to inform their responses for the sake of the next generation.

## Supplementary Information

Below is the link to the electronic supplementary material.

Supplementary Material 1

## Data Availability

Sample data is provided in the body of the paper and in the tables and figures. A full dataset is not available due to human ethics contraints.

## References

[CR1] Åkerlind, G. S. (2005). Variation and commonality in phenomenographic research methods. *Higher Education Research and Development*, *24*(4), 321–334. 10.1080/07294360500284672

[CR3] Aqtam, I., Shouli, M., Alqoroum, S., & Shouli, K. (2025a). Mental health interventions for resilience and mental health in conflict-affected Palestinian communities: A systematic review revealing research gaps and the absence of narrative therapy evidence. *Cambridge Prisms: Global Mental Health*, *12*(143). 10.1017/gmh.2025.10103 PMC12720377 41438269

[CR2] Aqtam, I. (2025). A narrative review of mental health and psychosocial impact of the war in Gaza. *East Mediterranean Health Journal*, *31*(2), 89–96. 10.26719/2025.31.2.89 40116261

[CR4] Aqtam, I., Ayed, A., Alfuqaha, O. A., & Shouli, M. (2025b). Stress and resilience of nursing students in clinical training during political violence: A Palestinian perspective. *PloS One*, *20*(6), e0325278. 10.1371/journal.pone.0325278 PMC12192045 40560858

[CR5] Aqtam, I., Naghnaghiyeh, R., Zubaide, T., Othman, Y., & Malasah, Y. (2025c). Psychological impact of political conflict: prevalence and severity of PTSD among children and adolescents in the north and middle West Bank following the october 7, 2023, events. *Nursing Research and Practice*, *1*, 7304673. 10.1155/nrp/7304673 PMC12349999 40810006

[CR6] Australian Curriculum (2010). https://www.australiancurriculum.edu.au/

[CR7] Barakat, S., Connolly, D., Hardman, F., & Sundaram, V. (2013). The role of basic education in post-conflict recovery. *Comparative Education*, *49*(2), 124–142.

[CR8] Chand, S. (2000). Coups, cyclones and recovery: the Fiji experience. *Pacific Economic Bulletin*, *15*(2), 121–129.

[CR9] Chanduvi, J. S., Jenkins, R., Dewan, P., Reuge, N., Yao, H., Alejo, A., & Bergmann, J. (2022). *Where Are We on Education Recovery*? *UNICEF*.

[CR10] Chen, J. J., & Krieger, N. J. (2023). Learning gain rather than learning loss during COVID-19: A proposal for reframing the narrative. *Contemporary Issues in Early Childhood*, *24*(1), 82–86. 10.1177/14639491211073144 PMC9988590 37520755

[CR11] Chichango, F., Cristóvão, L., & Mahanuque, O. (2023). Empowering women through vocational training: Evidence from rural areas affected by armed conflict in Mozambique. *Research Society and Development*, *12*(14), e108121441196–e108121441196.

[CR12] Cozolino, L. J. (2005). The impact of trauma on the brain. *Psychotherapy in Australia*, *11*(3), 22–25.

[CR13] Cozolino, L. (2014). *Attachment-Based Teaching: Creating a Tribal Classroom*. WW Norton & Company. The Norton Series on the Social Neuroscience of Education.

[CR14] Donnelly, R., & Patrinos, H. A. (2022). Learning loss during Covid-19: An early systematic review. *Prospects*, *51*, 601–609. 10.1007/s11125-021-09582-6 PMC8579897 34785823

[CR15] Engzell, P., Frey, A., & Verhagen, M. D. (2021). Learning loss due to school closures during the COVID-19 pandemic. *Proceedings of the National Academy of Sciences*, *118*(17), e2022376118. 10.1073/pnas.2022376118 PMC8092566 33827987

[CR16] Feuerstein, R., Feuerstein, R., & Falik, L. (2010). *Mediated Learning and the brain’s capacity for change*. Teacher’s College.

[CR17] Frankenberg, E., Sikoki, B., Sumantri, C., Suriastini, W., & Thomas, D. (2013). Education, vulnerability, and resilience after a natural disaster. *Ecology and society: a journal of integrative science for resilience and sustainability*, *18*(2), 16. 10.5751/ES-05377-180216 PMC4144011 25170339

[CR47] Friedman, H. H., Herskovitz, P. J., & Pollack, S. (1994). The biasing effects of scale-checking styles on response to a Likert scale. In *Proceedings of the American statistical association annual conference: survey research methods* (Vol. 792, pp. 792–795). https://rangevoting.org/FrDirBias.pdf

[CR18] Gebre-eyesus, M. T., & Reda, N. W. (2024). Tigray Education War Crisis: Implication for Sustainable Development Goals and Paths to Recovery. *Africa Education Review*, *20*(4), 18–32.

[CR19] Govender, I. (2023). Gender-based violence–An increasing epidemic in South Africa. *South African Family Practice*, *65*(3), 1–2. 10.4102/safp.v65i1.5729 PMC10091185 37042525

[CR20] Hajar, A. (2021). Theoretical foundations of phenomenography: a critical review. *Higher Education Research and Development*, *40*(7), 1421–1436. 10.1080/07294360.2020.1833844

[CR21] Harris, L. R. (2011). Phenomenographic perspectives on the structure of conceptions: The origins, purposes, strengths, and limitations of the what/how and referential/structural frameworks. *Educational Research Review*, *6*(2), 109–124. 10.1016/j.edurev.2011.01.002

[CR48] Hennink, M., & Kaiser, B. N. (2022). Sample sizes for saturation in qualitative research: A systematic review of empiric al tests. *Social science & medicine, 292*, 114523. 10.1016/j.socscimed.2021.114523 34785096

[CR22] Hlalele, D. (2012). Social justice and rural education in SA. *Perspectives in Education*, *30*(1), 111–118.

[CR23] Landa, N., Zhou, S., & Marongwe, N. (2021). Education in emergencies: Lessons from COVID-19 in South Africa. *International Review of Education*, *67*, 167–183. 10.1007/s11159-021-09903-z PMC8162486 34075259

[CR45] Maly, E., & Sakurai, A. (2023). Schools as Community Assets for Place-making in Post-disaster Resettlement: Reciprocal Impacts of Housing and Education Recovery in Tacloban City, the Philippines. In S. Huang & E. Maly (Eds) *Community Responses to Disasters in the Pacific Rim *(pp. 21–38). Routledge. 10.4324/9781003206415

[CR24] Marshall, N. (2014). *The Teacher’s Introduction to Attachment*. Jessica Kingsley.

[CR25] Martin, A. (2002). Motivation and Academic Resilience: Developing a Model for Student Enhancement. *Australian Journal of Education*, *46*(1), 34–49.

[CR27] Marton, F. (2004). Phenomenography: A research approach to investigating different understandings of reality. In *Qualitative research in education* (pp. 141–161). Routledge.

[CR26] Marton, F., & Booth, S. (1997). *Learning and Awareness*. Lawrence Erlbaum.

[CR44] Marzano, R. J., Kendall, J. S. (Eds.). (2006). The new taxonomy of educational objectives. Corwin Press.

[CR49] Miles, M.G., Huberman, A.M., Saldana, J. (2020). *Qualitative Data Analysis: A methods sourcebook*. Fourth edition. Sage.

[CR28] OECD (2025). *Education at at Glance 2025: South Africa*. https://www.oecd.org/en/publications/education-at-a-glance-2025_1a3543e2-en/south-africa_a1603bbe-en.html

[CR29] Opabola, E. A., & Galasso, C. (2024). Informing disaster-risk management policies for education infrastructure using scenario-based recovery analyses. *Nature Communications*, *15*(1), 325. https://www.nature.com/articles/s41467-023-42407-y 10.1038/s41467-023-42407-y PMC10770163 38182574

[CR30] Patterson, S. E., Verdery, A. M., & Daw, J. (2020). Linked lives and childhood experience of family death on educational attainment. *Socius*, *6*, 2378023120975594. 10.1177/2378023120975594 PMC8248584 34222657

[CR46] Plecher, H. (2020). South Africa: Urbanization in South Africa from 2009 to 2019 [online database]. New York, NY: Statista Inc. Retrieved 12 January 2021 from https://www.statista.com/statistics/455931/urbanization-in-south-africa/

[CR31] Psacharopoulos, G., Collis, V., Patrinos, H. A., & Vegas, E. (2021). The COVID-19 cost of school closures in earnings and income across the world. *Comparative Education Review*, *65*(2), 271–287.

[CR32] Seeger, M. W., Sellnow, T. L., & Ulmer, R. R. (1998). Communication, organization and crisis. In M. E. Roloff (Ed.), *Communication Yearbook* (p. 21). Greenwood Publishing Group.

[CR33] Shaw, J. A., & Harris, J. J. (2003). Children of war and children at war: Child victims of terrorism in Mozambique. In R. J. Ursano, C. S. Fullerton, A. E. Norwood (Eds) *Terrorism and disaster: Individual and community mental health interventions *41–57.

[CR34] SIMHA: Scalabrini Institute for Human Mobility in Africa (2026). South Africa. https://sihma.org.za/african-migration-statistics/country/south-africa

[CR35] Smith, M. (2021). *The Connected Therapist: Relating Through the Senses*. Author.

[CR36] South African Curriculum (2021). https://www.education.gov.za/Curriculum/NationalCurriculumStatementsGradesR-12.aspx

[CR37] Spector, M., & Kitsuse, J. I. (2017). *Constructing social problems*. Routledge.

[CR38] Srivastava, P., Cardini, A., Anderson, S., Bhatty, K., Gagnon, A., Jenkins, R., Matovich, I., Moriarty, K., Neuge, N., & Séguin, T. (2021). Rebuilding education systems for recovery: a crisis-sensitive and equity focused approach. *Policy brief Task Force 6: Social Cohesion and the Future of Welfare System*s, T20. https://www.global-solutions-initiative.org/wp-content/uploads/2025/03/TF6-REBUILDING_EDUCATION_SYSTEMS_FOR_RECOVERY_%E2%80%93_A_CRISIS-SENSITIVE_AND_EQUITY_FOCUSED_APPROACH.pdf

[CR41] UNESCO (2023). *Mozambique: Education Country Brief*. https://www.iicba.unesco.org/en/mozambique

[CR39] United Nations (n.d.) Sustainable Development Goals. https://sdgs.un.org/goals/goal4#overview

[CR40] United Nations Girls’ Education Initiative (UNGEI) (2024). School-Related Gender-Based Violence: Case Studies from Mozambique, Sierra Leone and Zimbabwe. https://www.ungei.org/sites/default/files/2024-12/UNGEI_SRGBV_CASE_STUDIES.pdf

[CR42] Van der Berg, S. (2008). Poverty and education. *Education policy series*, *10*(28), 1–28.

[CR54] Willis, A. (2021). Teachers’ cultural, social and emotional capabilities: how teacher compassion and humility is an antecedent to student confidence. *Pedagogy, Culture & Society, 31*(1), 91–108. 10.1080/14681366.2021.1884122

[CR51] Willis, A.S. (2018). The efficacy of phenomenography as a cross-cultural methodology for educational research. *International Journal of Research & Method in Education, 41*(5), 483–499. 10.1080/1743727X.2017.1283398

[CR52] Willis, A.S. & Nagel, M.C. (2015). The role that teachers play in overcoming the effects of stress and trauma on children’s social psychological development: evidence from Northern Uganda. *Social Psychology of Education, 18*(1), 37–54. 10.1007/s11218-014-9282-6

[CR53] Willis, A., Grainger, P., Menzies, S., Dwyer, R., Simon, S., & Thiele, C. (2021). The role of teachers in mitigating student stress to progress learning. *Australian Journal of Education, 65*(2), 122–138 10.1177/0004944120982756

[CR50] Willis, A., Devi, A., & Whitfield, H. (2025). Efficacious learning strategies and experiences for education recovery after disruption. *International Journal of Educational Development, 113*, 103198. 10.1016/j.ijedudev.2024.103198

[CR43] Zysberg, L., & Schwabsky, N. (2021). School climate, academic self-efficacy and student achievement. *Educational Psychology*, *41*(4), 467–482.

