# Supplementary material for: Education Recovery In Conflict-Affected Settings
Source: J Child Adolesc Trauma. 2026 Jul 14;19(4):3059–76. doi: 10.1007/s40653-026-00944-w (PMC13737325; doi:10.1007/s40653-026-00944-w)
Supplement: Supplementary file 1 [file 40653_2026_944_MOESM1_ESM.docx]

**Appendix 1: Interview Codebook**

| Codes and sub-codes (n=47) organised into categories of description | Participants (n=30) | References |
| --- | --- | --- |
| **Disruptions to education** |  |  |
| Poverty and lack of finances | 11 | 27 |
| No reading glasses | 1 | 5 |
| Start school late or missed time in school | 10 | 19 |
| No places in school or university | 2 | 4 |
| Death or sickness in family | 9 | 15 |
| Abuse, violence and injury | 9 | 14 |
| Gender based violence | 6 | 9 |
| COVID | 8 | 20 |
| Stress and trauma | 7 | 19 |
| Moving town or school | 6 | 8 |
| Long commute or rural isolation | 5 | 8 |
| Pregnancy and children | 5 | 9 |
| Civil unrest and violence | 4 | 11 |
| Parent or family obstructions | 3 | 4 |
| **Motivations** |  |  |
| Goals, dreams, determination or push | 14 | 40 |
| Providing for family | 7 | 11 |
| Enjoyment | 4 | 5 |
| Higher purpose or call | 4 | 7 |
| **Outcomes of disruptions** |  |  |
| Losses and gaps |  |  |
| Confidence | 6 | 6 |
| Knowledge and skills | 11 | 19 |
| Illiteracy | 3 | 9 |
| Life skills | 2 | 4 |
| Using story to help others | 6 | 7 |
| Hope | 5 | 10 |
| Turning to abusive relationships and prostitution | 5 | 9 |
| **Supports for recovery** |  |  |
| Salvation Army support | 11 | 17 |
| Supervisor or educator support | 10 | 10 |
| Workplace support and learning | 9 | 16 |
| Family support | 6 | 16 |
